# Dance for the dead: The role of top-down beliefs for social cohesion and anxiety management in naturally occurring collective rituals

**DOI:** 10.1371/journal.pone.0291655

**Published:** 2024-03-21

**Authors:** Briar Irving, Christopher Kavanagh, Ronald Fischer, Masaki Yuki

**Affiliations:** 1 School of Psychology, Victoria University of Wellington, Wellington, New Zealand; 2 School of Anthropology & Museum Ethnography, University of Oxford, Oxford, Oxfordshire, United Kingdom; 3 Department of Contemporary Psychology, Rikkyo University, Tokyo, Japan; 4 Institute D´Or for Research and Education, Rio de Janeiro, Brazil; 5 Department of Behavioral Sciences, Hokkaido University, Sapporo, Hokkaido, Japan; Utah State University, UNITED STATES

## Abstract

Collective rituals, particularly those characterized by synchrony and pain, have been shown to yield positive social and emotional outcomes. The question arises as to whether these findings extend to low-arousal, family-centered rituals and how spiritual beliefs factor into these communal practices. This study set out to examine the interplay between belief, ritual participation, and their effects on anxiety, social cohesion, and prosocial behavior during a low-arousal collective ritual in Mikasa, Japan. Drawing upon a sample of 183 festival participants, we measured belief in ancestors using a novel scale, identifying significant and consistent associations between these beliefs and measures of social cohesion across multiple targets. Moreover, active participation as a festival dancer displayed a positive relationship with feelings of social cohesion, particularly towards other festival attendees and at the national level. On measures of prosocial behavior, ancestral beliefs were positively associated with generosity shown within the festival setting, whereas observers were less generous towards community members than a non-attending control group. Anxiety outcomes displayed a negative relationship with ancestral beliefs and ritual observation but not participation as seen in previous research, suggesting a complex interplay between rituals, emotions, and individual states. These findings provide novel insights into the importance of belief systems and active participation in shaping social bonds and behaviors in the context of collective rituals.

## Introduction

Collective rituals are ubiquitous. Across the globe, people come together as communities to practice rituals, for births, deaths, marriage, the changing of the seasons, and a multitude of other reasons [1, 2]. These rituals play an important role in affirming cultural norms, increasing social cohesion, and managing emotional processes [3–7]. Yet despite the ubiquity of ritual and their enduring interest for anthropologists and sociologists, rituals have only recently begun to receive sustained focus in terms of psychology and quantitative explorations.

To date, much of the current experimental and field research has focused on the functional elements of the rituals, that is the behavioral components that may produce these effects via bottom-up processes [4]. For example, synchronized behavior increases social coordination and positive affect, while decreasing innovation, likely through shared attention mechanisms [8–10] and repetitive action decreases anxiety, likely via attention capture and motoric energy expenditure [11–14].

This line of research, therefore, prioritises actions and movement as a core functional element for ritualistic outcomes in a bottom-up fashion that does not require higher cognitive beliefs or meaning systems. The mere behavioral action seems sufficient for generating some degree of social cohesion [9]. Thus, individuals actively participating in ritualistic actions, compared to mere spectators, or other individuals not participating in rituals, would thus be expected to display higher social cohesion responses and lower anxiety. However, Hobson et al. [4] also highlighted the significance of top-down process associated with meaning and belief systems which may be equally important for producing ritualistic effects. This paper aims to contribute to related ongoing discussions on whether the social effects of rituals are focused on participants or generalize to other superordinate social categories [10, 22–25].

In our study, we focus on one large collective ritual performed annually in Japan, which involves specific beliefs surrounding ancestral spirits and therefore provides an opportunity to explore the role of both beliefs and ritual performance in the production of bonding, prosocial, and anxiety reducing effects. The Bon Festival, also known as Obon, is a significant holiday and ceremonial event in Japan that revolves around commemoration of the ancestors, who are believed to return to revisit family altars and graves during this period. The festival has syncretic origins related to traditional ancestral beliefs related to Shinto, Buddhist customs to honor the dead, and Confucian traditions.

The Buddhist aspects are traced back to celebrations derived from the Yulanpen Sutra, which recounts the tale of Mokuren, a disciple of the Buddha, who visualizes his deceased mother’s suffering in the afterlife and seeks the Buddha’s guidance for her relief [15]. Within Japan’s non-exclusive, syncretic, and orthopraxic religious environment [16, 17] the Bon Festival manifests as a mixture of religious traditions, incorporating aspects of Buddhism, Shinto, and local folk practices [18]. Here, the emphasis is more on the correct performance of ritual acts, reflective of Japan’s orthopraxic tendencies, rather than a strict adherence to a particular belief system [19].

A central feature of the Bon Festival is the Bon dance, or "Bon-Odori". The dance, which varies regionally, is a form of communal performance where participants circle a central platform, guided by rhythmic beats and songs. The songs often recount local histories, folklore, and events, and can serve as emblematic representations of a community’s shared memory [20]. While fundamentally celebratory, the dances and celebrations are often organized by temples and shrines, invoke religious iconography, and purport to help connect the participants with their ancestors. This mixture of festivity and reverence is emblematic of the Japanese religious landscape, where traditions seamlessly intersect, and practices often take precedence over any requirement to endorse specific doctrinal beliefs. There are typically no membership requirements or expressions of faith required to participate in the dancing, although the logistic arrangements for the festivals do involve a variety of formal groups and organizations. In general, the religious environment in Japan for the majority of the population does not involve regular attendance at religious services [17] but rather revolves around occasional participation in specific life stage rituals and annual celebratory festival (*matsuri*) events [19, 21]. Self-identification as a religious person is relatively rare in Japan (typically 10–15% in national surveys) and most rank religion as being of little importance in their life, however when measured via endorsement of broader supernatural beliefs, religious background, and participation in festivals and ritual celebrations there is significant religiosity or at least activities related to religious institutions and supernatural beliefs in Japan [17].

### Meaning systems: Ritualistic top-down effects

Hobson et al. [4] developed a framework of ritual in which they differentiated bottom-up processes from top-down, cognitive processes that involve ‘meaning creation and transference’. Ritual traditions attach meaning to a set of behaviours, which is passed on or shared with those performing the behaviours. Bottom-up processes of rituals involve the physical and automatic aspects of participation in a ritual, such as synchronous movement and biased attention through ritual performance, without requiring the activation of reflective conscious belief systems. Top-down processes on the other hand are driven by explicit cognitive processes, such as signalling social intentions and sharing meaning. Hobson and colleagues suggest that meaning transference regulates emotions and social connections by creating feelings of self-transcendence which alleviates anxieties, by reinforcing the perceived value of cultural knowledge and norms [see also 5]. They also suggest that meaning is a necessary feature of ritual, and without it a ritual would just be “arbitrary and trivial” [4, p. 261]. This proposed distinction parallels previous theoretical distinctions between intuitive ‘cognitively optimal’ elements of religious systems [26, 27] and those which are more elaborate, culturally constructed and hence ‘cognitively costly’ [28, Chapters 2 & 3]. Yet, we do not know much about the specific effects of top-down meaning attribution processes on ritualistic outcomes.

### Top-down effects of rituals on emotion regulation

A long line of research has demonstrated that ritualistic actions can help to regulate emotional processes, in particular down-regulating anxiety, and upregulating positive emotional responses [for ethnographic and conceptual discussions: 4, 29, 30; for empirical research: 12, 13, 25]. In addition, top-down processes are likely to play a role in emotional regulation, especially when considering the differential patterns across ritualistic practice [31]. A study by Brooks et al. [32], demonstrated that merely labelling arbitrary actions as rituals can increase the anxiolytic effects of repetitive actions. Similarly, a long line of research has pointed to positive associations between religious and spiritual beliefs and higher mental well-being, including decreased anxiety [33, 34]. These patterns in combination suggest that the subjective interpretation and associated expectations connected to ritualistic behaviour are playing a role in the reduction in anxiety.

### Contrasting bottom-up and top-down effects of rituals on social cohesion

The ability of collective rituals, especially when involving synchronized actions, to contribute to feelings of social cohesion is now well documented [6, 9, 22, 35–37]. We examine whether beliefs in the supernatural explanations attached to the ritualistic action will increase these effects. To the extent that belief systems form part of the identity of an individual [38, 39], we would predict that cohesive effects will be stronger for those who believe in the supernatural meanings attached to the ritualistic actions.

Another open question is the extent to which participating in collective rituals increases parochial or generalized prosocial tendencies. Evolutionary dynamics would suggest increased social bonding among coparticipants [40], however a number of studies show increases in social bonding beyond the immediate in-group after participating in ritual [10, 24, 25]. Therefore, in order to more clearly delineate these effects on social cohesion across levels, we examine social cohesion effects towards other participants in the ritual, towards the general community, and the nation as a superordinate category, separately in those who actively participated, those that observed and a control group.

We study the Bon Festival, a large-scale collective ritual celebrated annually in Japan, which is related to the Chinese Ghost Festival. Held in mid-August, the festival is a time when Japanese people return to their family home to welcome their family ancestors back to the home through various rituals and activities, including cleaning grave sites, decorating the household altar, and participating in Bon dances.

The Bon dance is a group dance conducted around a central platform; participants perform a short set of simple movements in synchrony to repeating folk song music. The dance moves performed, and songs played demonstrate regional diversity, yet there are a common core set of movements and strong consistency of style throughout Japan. The ritualistic focus on inviting and pleasing ancestors supposedly visiting the location during the ritual provides a useful opportunity to study the possible impact of relevant supernatural beliefs.

We use a new scale measuring ancestor belief in a quasi-experimental field study to compare the relative effects of supernatural beliefs vis-à-vis a) active participation, b) observation and c) a control group collected before the festival on social cohesion and anxiety responses. Importantly, even though it is an ancestor festival, there is wide variation in the extent to which the participants endorse beliefs in the supernatural components of the festival, regardless of whether they participate in the ritualistic actions [41–44]. This reflects a prevalent pattern in Japan and other East Asian sociocultural contexts, which have been described as ‘orthopraxic’ [45, 46], meaning that an emphasis is placed on practices rather than beliefs [17]. Therefore, the cultural context of the Bon festival allows us to survey people with an anticipated variety of belief levels who are participating to various degrees in a collective ritual in a naturalistic setting. Following previous research, we propose that belief will interact with participation, where higher levels of belief will increase the effects of participation, leading to reduced anxiety and higher social cohesion outcomes.

### Ethics

The study design was approved by the School of Psychology Human Ethics Committee delegated from the Victoria University of Wellington’s Human Ethics Committee (Approval # 0000026999) and by the Central University Research Ethics Committee at Oxford University (Ref: SAME_C1A_16_015). All participants provided written consent to participate, and data was anonymized prior to analysis.

### Participants and design

We conducted a quasi-experiment during the Bon dance at a Bon Festival in Mikasa, Hokkaido, in Japan. A convenience sample of 196 members of the Mikasa community and visitors from the surrounding area to the festival participated. Respondents were approached either at the festival (observers and dancers), or at a local supermarket (control), and asked if they would like to participate. Survey questions differed slightly between groups: observers and dancers were asked one extra set of cohesion and dictator questions for “other participants” that the control group were not asked due to contextual differences. 183 responses were valid, of these, 43 were in the control group, 90 were observers, and 50 were dancers (see Table 1 for more details). As a power analysis for our planned analytical methods suggested a minimum of 150 participants, we were content with this sample size. Ages ranged from 18 to 82 with a mean age of 63 (SD = 12.66), 40 (SD = 14.94), and 46 (SD = 14.47) for the control group, observers, and dancers respectively. Gender was relatively evenly split with 57% female and 42% male overall (Control group: 65:34, Observers: 56:43, 1% other, Dancers: 48:52), 1% answered as other. 50% of participants currently resided in Mikasa (Control group: 84%, Observers: 39%, Dancers: 42%), 13% lived in the nearest large town, Iwamizawa (Control group: 5%, Observers: 18%, Dancers: 10%), and 37% were from outside of those areas (Control group: 12%, Observers: 43%, Dancers: 48%). After excluding 13 responses for incomplete surveys in which substantial sections of the survey were missing, the small amount of remaining missing data was checked for randomness and imputed using Multivariate Imputation By Chained Equations (MICE) [47].

**Table 1:** Demographics.

Group	Age M (SD)	Residence Mikasa%, Iwamizawa%, Other%	Ancestral Belief score M (SD)	Supernatural Belief Score M (SD)
Control	63.16 (12.66)	83.7%, 4.7%, 11.6%	2.45 (0.87)	3.09 (1.12)
Observers	40.29 (14.94)	38.9%, 17.8%, 43.3%	2.72 (0.77)	3.14 (1.02)
Dancers	46.20 (14.47)	42.0%, 10.0%, 48.0%	2.75 (0.76)	3.09 (1.01)

### Age differences in sample groups

Looking at our demographic variables, it was clear that the control group was significantly older than the field sample, t(184) = -8.31, p < .001. As such we performed a correlation analysis to identify which results may be affected by this difference. We found six significant correlations with age: Identification with community (r = .15, *p* = .04), frequency of grave cleaning (r = .35, *p* < .001), returning to the family home for Bon (r = -.20, *p* = .02), donation to community (r = .25, *p* < .001), donation to national charity (r = .22, *p* = .003), and the Supernatural Belief Scale (r = -.18, *p* = .02). However, when we performed a series of regressions controlling for whether the participant came from the control or the field sample, the effect of age became non-significant for predicting identification with community, returning to the family home for Bon, donation to community, and donation to national charity. The only two outcomes for which age remained a significant predictor were grave cleaning and the Supernatural Belief Scale. This suggests that regardless of the sample, older people perform grave cleaning more often, and had slightly lower endorsement of supernatural beliefs. The first of these is unsurprising as older people are more likely to both have the free time required to regularly perform grave cleaning and are more likely to have strong connections with now-deceased family members. The slightly lower supernatural belief is unexpected as the general trend is for younger people to have lower religious adherence [48]. However, it should be noted that the mean scores differed between the control sample and both observers and participants at a magnitude of less than half a point on a 5 point response scale.

### Materials

#### Dependent variables

*Anxiety*. We measured anxiety using a modified version of the State-Trait Anxiety Inventory–Trait Short Form [49]. This is a 5-item measure rated on a 4-point Likert scale from 1—“全くそう思わない” (not at all) to 4—“強くそう思う” (very much so), with a higher score indicating more anxiety (Japanese version by Koizumi, Fujita, Ninomiya, and Nakamoto [50]. We found during testing that the inventory had low internal reliability (α = .66), which was due to low intercorrelations of items 2 and 4. When these items were removed internal reliability increased to a more acceptable level (α = .85). Due to the low reliability metrics, in the subsequent analysis, the results reported are for the 3-item version of the inventory.

#### Social cohesion

Social cohesion was assessed using two self-reported scales: a 5-item version of the identity fusion scale by Gómez et al. [51], slightly adjusted for clarity in Japanese and a 4-item measure of social identification adapted from Leach et al.’s [52] and Postmes, Haslam, and Jans’ [53] work. Both scales were measured on response scales from 0 to 4, with higher scores indicating higher levels of fusion or identification. Participants answered the fusion and identification items for three target groups: 1) coparticipants in the festival ritual; 2) the local community, 3) Japan. The coparticipant target was not included for the control group as they were not at a festival. However, despite intentions to treat these scales as separate constructs a CFA (see S1 File) indicated that in this sample the high level of correlations meant that a single-factor model was better supported than the anticipated two-factor model separating fusion and identification. As a result, we combined the 5-item fusion and 4-item identification scales into a single 9-item *social cohesion* measure with a score for each of the three target groups: national (α = .95), community (α = .96), and festival participants (α = .94). Scale metrics revealed strong internal reliability for each target.

#### Prosocial behavior

To measure a prosocial group behaviour, we examined participants allocation choices in an economic game. Specifically, we used a variation of a dictator game, in which individuals had to allocate 10,000 JPY (around 100 USD) between themselves and a given group, which varied in level of local proximity between rounds (e.g., another unknown participant, community level, national level). Respondents were given the option to enter a draw to have one of their choices paid out (to both self and the respective recipient group). All individuals made allocation choices, but only 40% of participants chose to enter the draw to receive a payout.

#### Ancestral belief scale

We developed a new scale to measure belief in ancestral spirits (see S1 File for details). The scale was developed based on an extensive literature search of ethnographic studies, and a series of focus groups with 6 participants. A pilot study (N = 151) with 17 items showed good internal reliability (α = .96) and good convergent and discriminant validity (see S1 File). We used the five highest loading items from the pilot test, with example statements being “During bon, my ancestors travel home” and “I feel like my ancestors are around me during bon”. Responses were recorded on a 1–4 Likert type scale. We also included a short version of the Supernatural Belief Scale [SBS; 54], measured on a 5-point Likert scale (ranging from -2 to +2) to insure divergent validity in the current example. The ancestral belief scale (α = .89), and the SBS (α = .91) demonstrated good internal reliability. They exhibited a moderately positive correlation (*r* = .35, *p* < .001).

#### Procedure

We surveyed our control group 2 weeks prior to the festival in a local supermarket. The participants and observer data were gathered on the two final days of the festival, at the festival location. Participants were identified as anyone at the festival, with ‘dancers’ those who had participated in the Bon dance ritual that night, prior to being surveyed, and observers those at the festival who had not participated in the Bon dance on that day and thus were, at the time of completing the survey, ‘observers’.

## Results

### Anxiety

**Fig 1:**
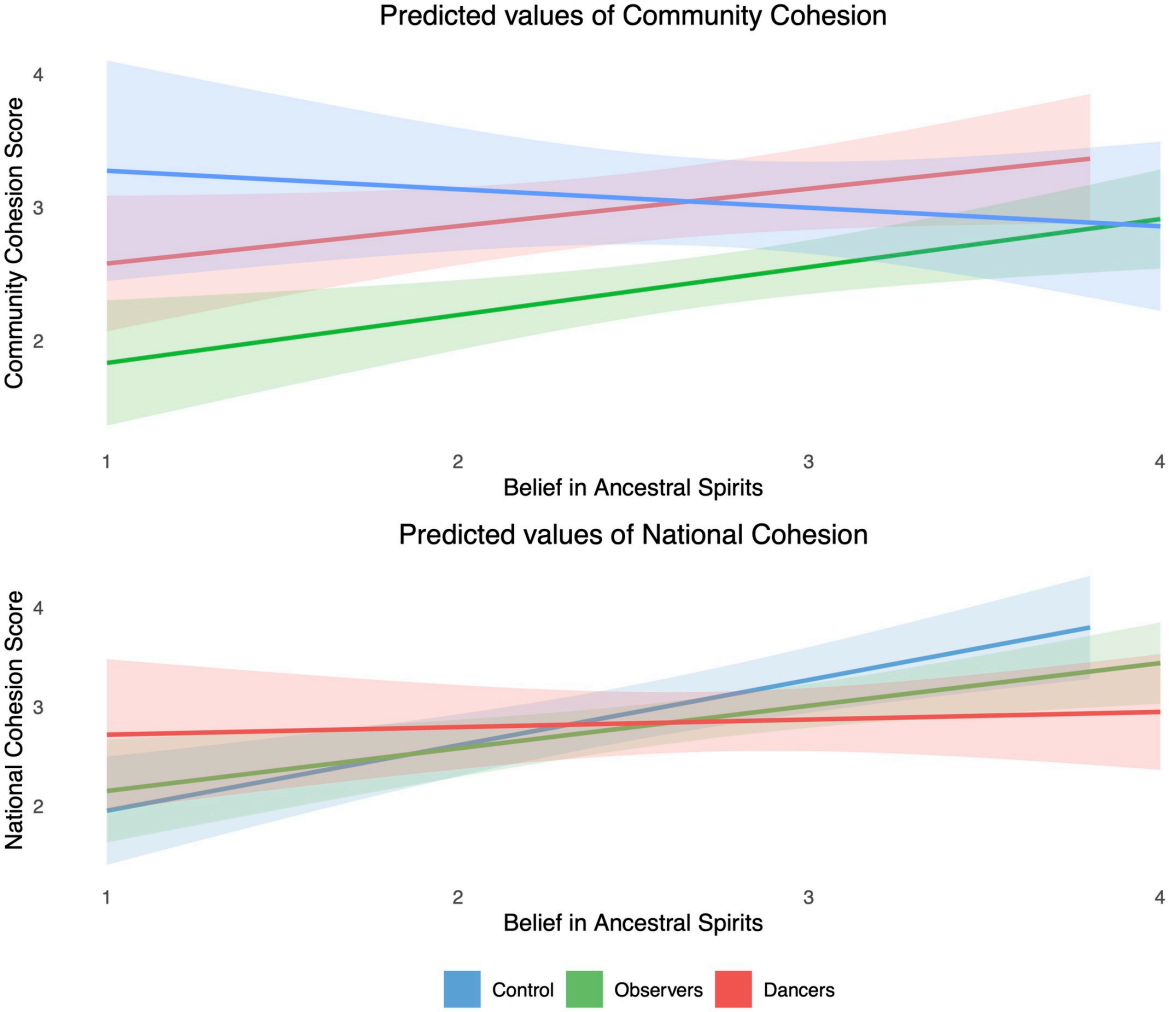
Distribution of anxiety scores by group.

**Fig 2:**
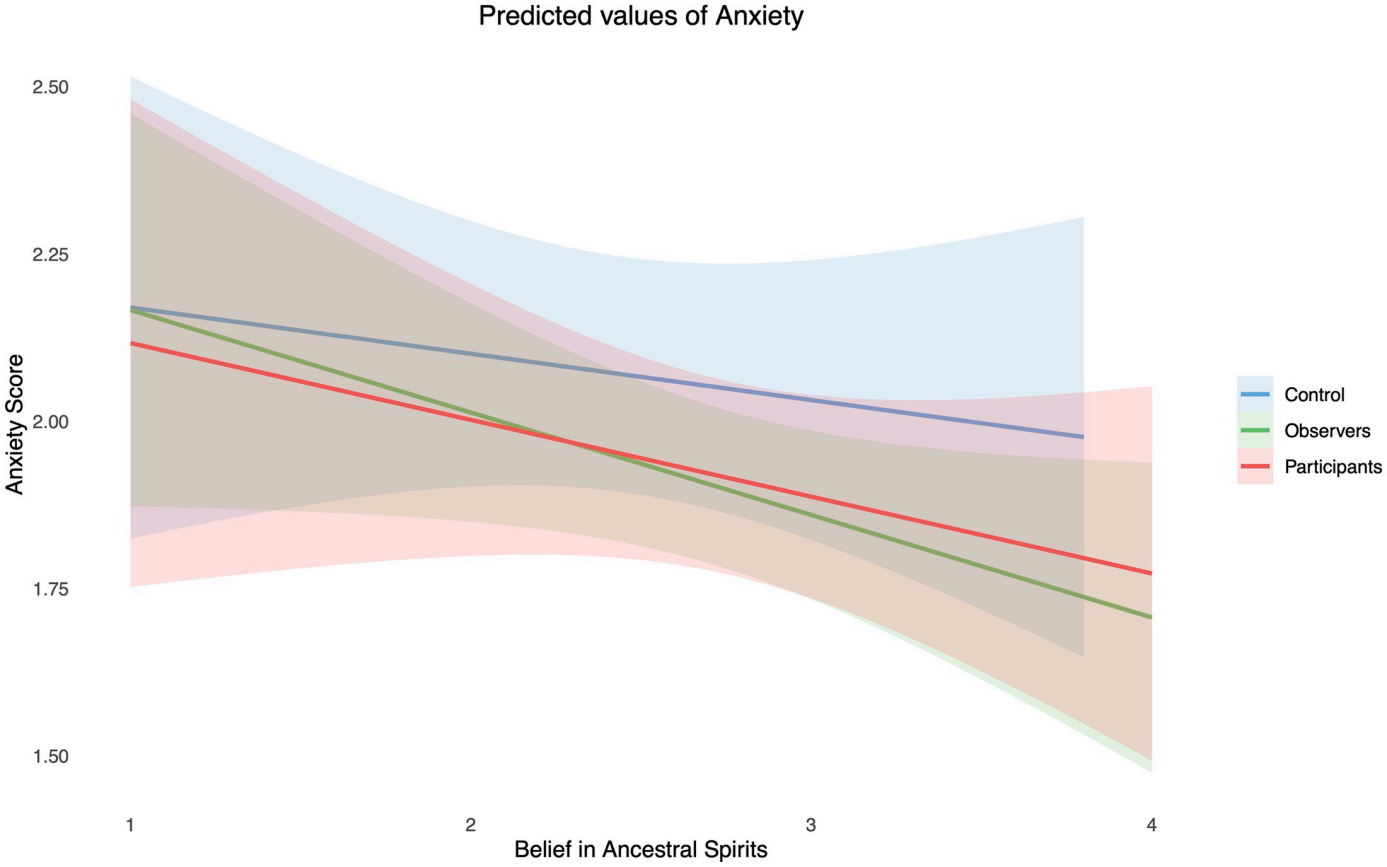
Predicted anxiety scores by belief in ancestral spirits.

Our regression model, which predicted anxiety from our dummy coded group variables, ancestral belief, and their interactions, was found to be statistically significant, F (7,175) = 2.15, *p* = .04, R^2^ = .08. We had anticipated main effects for belief and participation, but contrary to these expectations, neither belief (b = -0.05, *p* = .58) nor festival participation (Control vs Observers, b = -0.03, *p* = .92; Control vs Dancers, b = -0.22, *p* = .57) displayed significant relationships (see Fig 1 for distributions by condition). Additionally, the interactions we included in our model did not contribute significantly to predicting anxiety. However, when the interactions were removed a negative main effect was observed with ancestor beliefs (b = -.11, *p* = .004) (see Fig 2 for a visual representation), meaning that each single unit increase on the ancestral belief scale there was an associated decrease of 0.11 on the anxiety score (Table 2).

**Table 2:** Regression model with anxiety score as outcome.

Variable	B (SE)	p-value
**Intercept**	2.78 (0.25)	< .001
**Belief**	-0.11 (0.05)	.004
**Observer (vs. Control)**	-0.29 (0.12)	.02
**Dancer (vs. Control)**	0.23 (0.13)	.06
**Age**	-0.01 (0.003)	.02
**Gender**	-0.02 (0.08)	.80

Model Metrics: F (5, 177) = 2.85, *p* = .02, R^2^ = .07, Adjusted R^2^ = .05

### Social cohesion

Social cohesion was assessed with a series of regressions examining self-reported bonds at three different levels (participant, community, nation) while controlling for age and sex (Table 3).

**Table 3:** Regression models with social cohesion outcomes.

Outcome Target	1. Co-Participants		2. Community		3. National	
	B (SE)	Sig.	B (SE)	Sig.	B (SE)	Sig.
**Intercept**	1.13 (0.35)	.001	1.47 (0.56)	.01	0.31 (.57)	.59
**Belief**	.38 (.12)	.002*	.30 (.17)	.08	.66 (.17)	< .001***
**Observers (vs. 1: Dancer, 2–3: Control)**	1.24 (.04)	.03*	-.78 (.58)	.18	.52 (.59)	.38
**Dancers (vs. Control)**	—	—	1.25 (.68)	.07	1.58 (.70)	.03*
**Belief x Observers**	-.23 (.21)	.28	.08 (.21)	.71	-.22 (.22)	.32
**Belief x Dancers**	—	—	-.48 (.25)	.05*	-.65 (.25)	.01*
**Age**	-.001 (.01)	.92	.003 (.01)	.49	.01 (.01)	.19
**Sex**	.39 (.15)	.01*	.43 (.14)	.003**	.41 (.15)	.006**
**Model Metrics**	F(5, 134) = 7.33, p < .001		F(7, 175) = 5.24, p < .001		F(7, 175) = 4.96, p < .001	
R^2^	R^2^ = .22, Adj. R^2^ = .19		R^2^ = .17, Adj. R^2^ = .14		R^2^ = .17, Adj. R^2^ = .13	

In the context of social cohesion with other festival participants, we found a significant main effect of belief (b = 0.38, *p* = .002) and being a dancer as compared with being an observer (b = 1.24, *p* = .03). Belief in ancestral spirits was thus associated with a stronger feeling of connection with other festival-participants and those participating in the ritual dance displayed higher cohesion scores than observers. No interaction was observed between ancestor beliefs and festival role.

**Fig 3:**
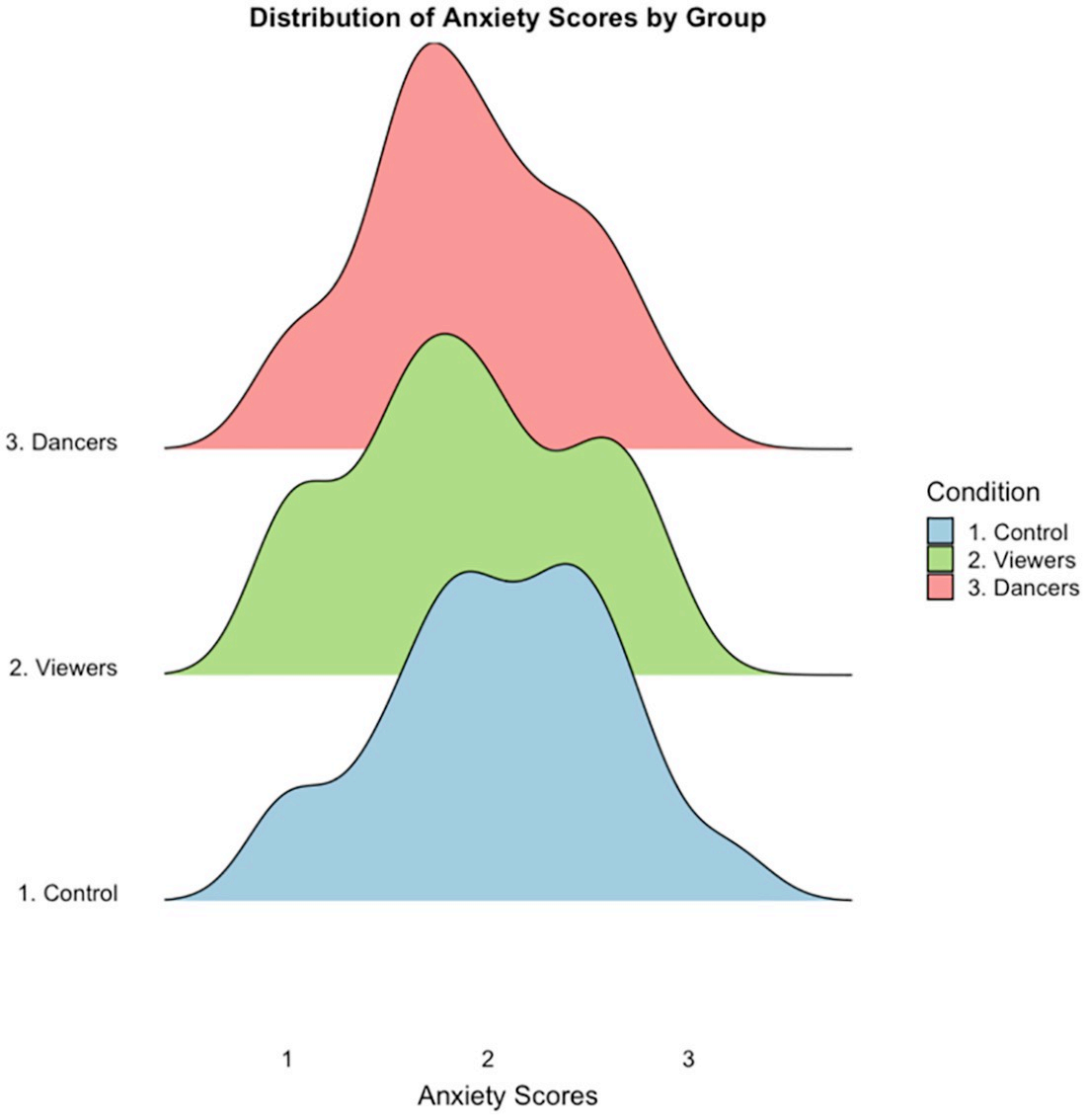
Predicted cohesion scores by ancestral spirit beliefs.

Regarding social cohesion with the community target, dancers (compared with control) displayed a positive association (b = 1.25, *p* = .07) but this was outside conventional significance thresholds. There was a main effect for belief observed but again it was not significant (b = .30, *p* = .08). However, there was a direct relationship (b = .21, *p* = .02) in a reduced model that included no interactions: F(5, 177) = 5.88, *p* < .001, R^2^ = .14. In the full model there was an interactive effect observed between participation as a dancer and ancestral belief, although this too was close to the significance threshold and should be interpreted cautiously (b = .48, *p* = .05). Fig 3 displays the predicted values of Community Cohesion scores against ancestor beliefs for the three different groups: Control, Observers, and Dancers. Each point on the plot represents the predicted value of community cohesion at different levels of ancestor belief. As can be observed from the regression lines with shaded confidence intervals, there is an anticipated positive relationship for observers and dancers and a negative relationship with the control group.

For the national target, a strong main effect of belief was found (b = .66, *p* < .001) and there was a positive association (b = 1.58, *p* = .03) with dancers as compared with the control group. A positive interaction was observed between participation as a dancer and ancestral beliefs (b = -.65, *p* = .01), though here it is important to note that the effect was driven by the control group where there was a strong correlation observed between national cohesion and ancestral beliefs (N = 43, *r* = .54, *p* < .001), whereas no such relationship was observed with dancers (N = 50, r = .06, *p* = .70). See Fig 3 for a visual representation of predicted values and note that the cohesion score for dancers is flat as compared to the positive predicted relationships for observers and the control group. Additionally, a model with the same predictors but no interactions entered: F(5, 177) = 5.43, *p* < .001, R^2^ = .13 found significant relationships only for sex (b = .39, *p* = .01) and ancestral beliefs (b = .40, *p* < .001).

Across all models, age was never a significant predictor, but the sex of the participants showed a consistent significant effect (Participant: b = 0.39, *p* = .01; Community: b = 0.43, *p* = .003; National: b = 0.41, *p* = .01), with males reporting stronger social bonds compared to females.

Overall, in terms of the interaction between bottom-up (ritual participation) and top-down processes (belief system), the regression models reported significant relationships for dancers with both community and national level social cohesion ratings. Belief in ancestors displayed a consistent positive relationship in all models without interaction terms, and with interactions included it displayed a consistent positive relationship, with only the community cohesion model falling outside significance thresholds, b = .30, *p* = .08.

### Prosocial behavior

In relation to prosocial behaviour in allocations in a dictator game, using the same predictors as with the social cohesion outcomes (Table 4), we found a main effect of belief on generosity towards other festival participants (b = 1.13, *p* = .01) but no other target (including in models with no interactions). Individuals who more strongly endorsed ancestral spirit beliefs displayed more generous allocations towards other festival participants. Contrary to previous research, as seen in Table 3, we found a negative main effect of observing the festival vs the control group in allocations for the community target (b = -4.02, *p* = .05), however it should be noted that this result was on the threshold of significance. The control group was more generous towards other community members compared to observers but there was no difference observed for festival dancers (b = -1.10, *p* = .93). If the interaction terms was removed from the community target model then there was a similar negative relationship observed: b = -3.36, p < .001, F(5,177) = 7.01, P < .001, R^2^ = 17. If we assume the effect is not a statistical artefact, the difference could be assumed to be due to the control group having a higher proportion of Mikasa residents than the festival observer group but the effect remained even after controlling for place of residence (b = -4.33, *p* = .03). There were no significant interactions found between belief and festival participation for community and national targets, but the interaction effect in the co-participant model was b = -1.48, *p* = 0.05.

**Table 4:** Regression models with prosocial behavior outcomes.

Outcome Target	1. Co-Participants		2. Community		3. National	
	B (SE)	Sig.	B (SE)	Sig.	B (SE)	Sig.
**Intercept**	0.04 (1.68)	.98	3.06 (1.95)	.12	4.21 (1.99)	.04*
**Belief**	1.13 (0.44)	.01*	0.41 (0.59)	.49	0.22 (0.60)	.72
**Observers (vs. 1: Dancer, 2–3: Control)**	3.43 (2.12)	.11	-4.02 (2.02)	.05*	-2.81 (2.06)	.17
**Dancers (vs. Control)**	—	—	-0.22 (2.39)	.93	0.30 (2.44)	.90
**Belief x Observers**	-1.48 (0.76)	.05	0.68 (0.75)	.37	0.41 (0.76)	.59
**Belief x Dancers**	—	—	-1.10 (0.86)	.20	-1.36 (0.88)	.12
**Age**	0.02 (0.02)	.41	0.03 (0.02)	.14	0.03 (0.02)	.11
**Sex**	0.69 (0.54)	.21	1.17 (0.50)	.02*	0.41 (0.51)	.42
**Model Metrics**	F(5, 134) = 1.88, p = 0.10		F(7, 175) = 5.83, p < .001		F(7, 175) = 5.23, p < .001	
R^2^	R^2^ = .07, Adj. R^2^ = .03		R^2^ = .19, Adj. R^2^ = .16		R^2^ = .17, Adj. R^2^ = .14	

## Discussion

We tested the effects of top-down belief systems on levels of social cohesion, prosocial behavior, and anxiety outcomes in one large scale collective festival featuring ritual dances vis-à-vis bottom-up participation effects comparing active ritual participants (dancers), observers of the ritual, and a non-observing control group. First, we found a consistent positive association between level of stated belief in ancestor spirits, using our novel ancestral belief scale, and our social cohesion measures across all three target groups. This suggests that such beliefs may play a significant role in fostering social bonding.

Active participation in the festival as a dancer displayed a similar positive relationship with feelings of social cohesion, particularly with other festival participants and at the national level. This relationship was more robust when examined in models without interactions but should still be interpreted with more caution as in the full models the relationships observed were just within (co-participant target, b = 1.24, *p* = .03; national target, b = 1.58, p = .03) or just outside conventional significance thresholds cohesion: community target (b = 1.25, *p* = .07).

When exploring the interaction between belief and participation, we observed a negative relationship with the community (b = -.48, *p* = .05) and national targets (b = -.65, *p* = .01). Examination of these interactive relationships (see Fig 3) suggested that community cohesion scores increased with belief in ancestral spirits for dancers and potentially observers whereas the opposite relationship was observed with the control group. Conversely, with the national target enhanced ancestral belief scores made no difference to dancers but there was a positive association for the control group respondents and observers.

These findings highlight the complex ways in which cultural practices and beliefs can influence social bonds. It’s clear that both belief in ancestral spirits and active participation in the festival are important, but the nature of their effects can vary depending on the specific social context (i.e., participant, community, or national level) and the nature of an individual’s level of participation in the festival. This accords with previous studies that have noted differential impacts on ritual participants vs. observers.

Overall, then in our data, self-reported endorsement of ancestral beliefs displayed a positive relationship with social cohesion, though the relationship was weakest with the community target. Active participation in the ritual dances also displayed a positive association but the relationship was weaker than observed with ancestral beliefs.

Results on our measure of prosocial behaviour based on dictator game allocations were less consistent. Ancestral beliefs did display a relationship with donation to festival co-participants (b = 11.13, *p* = .01) and there was some evidence of an interactive effect between participation and belief though this was on the threshold of significance (b = -1.48, *p* < .05). This suggests that individuals who express a stronger endorsement of ancestral spirit beliefs, in turn, exhibit greater generosity within the festival setting. This relationship illuminates the potential influence of deeply held cultural beliefs on prosocial behavior within a context of shared social activity.

Contrastingly, observers of the festival demonstrated less generosity towards community members compared to the control group (b = -4.02, p = .05). While this finding is on the edge of significance in the full model, it is notable that the negative relationship remained upon removal of the interaction terms from the model (b = -3.36, p < .001) and even after controlling for the place of residence (b = -4.33, p = .03). This may imply the control group’s higher proportion of Mikasa residents served as a contributing factor, though notably no such relationship was observed with dancers and community donation allocations. Furthermore, in terms of interaction effects between belief and festival participation, none were found for community and national targets.

Overall, the relationships revealed in the prosocial behaviour analysis are less robust than those found with social cohesion measures. This discrepancy may stem from limitations in the measurement method, notably only 40% expressed interest in receiving the payout which might speak to reduced involvement, or alternatively simply that self-reported feelings are more prone to immediate environmental influences than monetary behaviors. Additionally, it is inevitable that the dynamic and distraction-rich festival environment may have impacted the attentiveness of participants.

Our allocation task findings contrast with reported results of greater prosocial behavior following actions involving synchrony and shared intentionality, as measured with cooperative behavior in a public goods game [10] or volunteering to donate time [24]. An important difference is that these experiments were conducted in artificial laboratory conditions with naïve subjects in English speaking countries and were not targeting pre-existing social groups. In such abstract and controlled contexts, a synchrony manipulation has the potential to produce more substantial impacts than would be the case in a field setting with the additional confounds that it entails. Moreover, as our control group resided in the relevant community, they may have felt some social obligation to reassert their community membership when presented with an allocation task from non-local researchers. Further research is necessary to investigate whether comparable effects can be observed in laboratory and field conditions.

In relation to previous findings that ritual decreases anxiety, we did not find any interactive relationship but when we examined a model looking only at main effects we found both ancestral beliefs and observing the festival to be anxiety reducing. Conversely, festival participation indicated a positive though non-significant relationship with anxiety levels (b = .23, *p* = .06). One point to note here is that anxiety levels in our population were very low, possibly leading to floor effects. Further, ritual participation may show anxiolytic effects, but only if participants are in an elevated anxious state [see 12, 55]. It is also likely that certain rituals will be anxiety promoting in the short term but alleviating or mentally reassuring over time. Future studies may want to focus on different rituals that involve more elevated anxiety states if they want to explore this relationship [see 14].

Our study is limited by the fact that it is a quasi-experimental study, without the power to infer causation, and that it was conducted in situ. This means the ecological validity of the study is improved but inevitably internal validity is reduced as the field setting introduces a host of confounding environmental and logistical factors. Additionally, our control group was significantly older than the participant and observer groups, and while we did not find evidence from our control measures that this had a significant effect on our results, we would still caution against overinterpreting the comparisons with our control group. Future studies should more carefully match participants in the control and ritual conditions, or better, use a within subjects’ longitudinal study with each person serving as their own control to properly address these issues.

## Conclusion

Our research provides novel insights into the interplay of belief systems, festival participation, and social dynamics by examining their influence on social cohesion, prosocial behavior, and anxiety outcomes in the context of a large-scale festival characterized by ritual dances. We observed that endorsement of ancestral beliefs was consistently associated with increased social cohesion across multiple target groups, indicating the potential role such beliefs play in fostering social bonding.

Similarly, active participation as a festival dancer was found to have a positive relationship with feelings of social cohesion, especially towards other festival participants and at a national level. Despite some findings being on the threshold of significance, these patterns underline the importance of participation in social rituals for enhancing social bonds.

We also observed complex interaction effects between belief and participation on social cohesion, highlighting how cultural beliefs and social context are intricately intertwined. The nuanced effects suggest that both belief in ancestral spirits and active participation in cultural festivals are important, but their impacts vary depending on specific social contexts and individual’s level of participation in the festival, echoing previous research that identified differential impacts on ritual participants versus observers.

In terms of prosocial behavior, ancestral beliefs exhibited a positive relationship with generosity, particularly within the festival setting. However, observers were less generous towards community members than the control population. These results, although less robust than the social cohesion findings, provide insights into the dynamics of prosocial behavior in a real-world setting.

Out study echoes previous research that both belief and ritual observation can alleviate anxiety, however in our data there was also a potential anxiety inducing relationship with ritual participation. This highlights the complex interplay between ritual forms, emotions, and individual states, and suggests further research on a broader range of rituals and over longer timeframes may be needed.

While our study’s quasi-experimental design in an ecologically valid setting presented challenges and limitations, the findings underline the vital roles of belief systems and active participation in shaping social bonds and behavior. Our results remind us of the richness and complexity of festival and ritual experiences in natural settings and contribute to developing a more nuanced understanding of the psychological processes unfolding during communal rituals. Future research should further explore these multifaceted dynamics to fully uncover the psychological impacts of collective rituals and cultural beliefs.

## Supporting information

S1 File (DOCX)
