# Supplementary material for: Dance for the dead: The role of top-down beliefs for social cohesion and anxiety management in naturally occurring collective rituals
Source: PLoS One. 2024 Mar 21;19(3):e0291655. doi: 10.1371/journal.pone.0291655 (PMC10956785; doi:10.1371/journal.pone.0291655)
Supplement: S1 File — (DOCX) [file pone.0291655.s001.docx]

# Supporting information

In the initial stage of this study the first author developed six draft items for the belief scale based on ethnographic literature surrounding the Bon Festival and the Bon dance in particular [1-6].

The first item was developed to reflect the very basic essence of what happens during Bon, which is the main spiritual reason for holding the Bon and this is with all interpretations:

“This is the time for family reunions of the spirits of the dead and the living” – [5, p. 145]

“July 13-16 (August 27-30). Bon Festival. Members of branch families clean the ancestors' tombs (usually stone monuments) in the main family's graveyard on July 13. Early in the morning of July 14, members of branch families gather at the main family's house in order to perform the Bon Festival (memorial services for the spirits of ancestors and all souls of the dead); they clean the house and prepare the ornaments and new altars for the coming spirits or souls from “Other Land”.” – [1, p. 409]

A1. During bon, my ancestors travel home

お盆の時期には、先祖が家に帰る。

The next items were developed based on accounts in various ethnographic theology texts which described an increase in communication with ancestor spirits during bon. Most of this came in the form of prayer and offerings, but also physical acts such as carrying ancestors from their place of rest.

“The people of former times naturally spoke to ancestors as they did to living persons, calling them *jii-sama, baa-sama.* Recently only children say these words… but in the Chubu area with which I am familiar, people in mature years recall that when they went as children in the evening on the 13^th^ day to the grave… they put their hands behind their backs and “*jii-sama, baa-sama*, let’s start now”.” – [2, p. 139]

“We asked the question if the ancestors somehow, under one form or another, still lived on somewhere. The 67-year-old head of a first rank stem family, told us that he lived constantly in their presence. This same informant told us also that, every year at o-bon, he held the welcome fire as soon as possible and the sending-off fire as late as possible in order that the ancestors may stay longer. (Four months later at o-bon, when we checked this, it proved indeed to be true: he was at least two hours ahead of his neighbours for the welcome fire.)” – [6, p. 298]

“There was once a hardworking couple who did not rest at *Bon*, and as they continued to work in their garden, they heard somebody talking as he passed but could see no sign of him as he said, “I returned for this occasion, but they made no preparation for me. It made me so angry, I knocked him down.” Startled, the couple rushed home excitedly and found their baby had fallen into the open hearth and was hurt.” – [2, p. 140-141]

A2. It is easier to communicate with ancestors during bon.

お盆の時期には、先祖と話をしやすい。

And

A3. Ancestors are closer during bon than the rest of the year.

お盆の時期には、それ以外の時期と比べて、先祖が近くにやってくる。

Drawing on the same material as above, the following item was developed to capture the return to non-sacredness after the end of the festival.

A4. Ancestors leave again at the end of bon.

お盆が終わると、先祖はまた帰って行く。

In order to avoid response sets and demand characteristics, we included two reverse coded items based on these ethnographic sources.

A5. Ancestors do not return during bon.

お盆の時期に、先祖が家に帰ってくるわけではない。

And

A6. You cannot communicate more easily with ancestors during bon.

お盆の時期に、先祖と話がしやすくなるわけではない。

The 6 items developed from the ethnographic literature were then first discussed with **[Author Name]** to get an initial perspective on the appropriateness of the items in modern Japan and ensure they made sense. This brought up a number of questions which were brought to a focus group discussion, delving into the nuance of feelings around bon and how best to capture these beliefs in survey items. The focus group consisted of 5 postgraduate university students at Hokkaido University, 20% female, from both rural and city backgrounds throughout Japan.

During the focus group discussions, group members were asked whether each item was understandable, clear, and appropriate. To increase clarity, the following changes were made: Item A2 was changed to reflect that the communication was direct but one sided, and “easier” was removed; Group members discussing item A3 felt that the type of closeness to ancestors felt varied person to person so this item was changed to allow individual interpretation of physical or psychological closeness; Focus group members felt that item A6 was unclear and unnecessary so this item was replaced with a reverse code of A3; References to ancestors in all items were addended with “ ‘s spirit” to provide clarification and make the items sound more natural. New items developed with the help of the focus group, include “It is important to pray to ancestors during bon”, “Cleaning the family grave shows returning ancestors they are cared for”, “Ancestors reside in the butsudan during bon”, “It is important to provide offerings for ancestors during bon”, “My ancestors would be bothered if I didn’t pray to them during bon”, “I feel like ancestors are watching me during bon”, and “Ancestors can affect my fortunes during bon”. After this development we were left with a total of 17 items.

|  | **Bon Belief Scale - Full** | |
| --- | --- | --- |
|  | **English** | **Japanese** |
| 1 | During bon, my ancestors travel home | お盆の時期には、先祖の魂が家に帰る。 |
| 2 | I can talk to ancestors during bon | お盆の時期には、先祖の魂に話しかけることができる。 |
| 3 | I feel like ancestors are watching me during bon | お盆の時期には、先祖が自分を見ているような気がする。 |
| 4 | I feel ancestors are around me during bon | お盆の時期には、先祖の魂が自分の近くにいるような気がする。 |
| 5 | Ancestors leave again at the end of bon | お盆が終わると、先祖の魂はまた帰って行く。 |
| 6 | I feel like ancestors are physically close to me during bon | お盆の時期には、先祖の魂が自分の体の近くにいるような気がする。 |
| 7 | I feel like ancestors are psychologically close during bon | お盆の時期には、先祖の魂が自分の心の近くにいるような気がする。 |
| 8 | It is important to pray to ancestors during bon. | お盆の時期に先祖に祈ることは大切だ。 |
| 9 | Cleaning the family grave shows returning ancestors they are cared for | お墓を掃除すれば、先祖たちは自分たちが大切にされていることを感じることができる。 |
| 10 | Ancestors reside in the butsudan during bon | お盆の時期には、先祖の魂は仏壇の中にいる。 |
| 11 | My ancestors listen when I pray during bon | お盆の時期に私が祈ると、それは先祖の耳に届く。 |
| 12 | It is important to provide offerings for ancestors during bon | お盆の時期には、先祖にお供物を捧げることが大切だ。 |
| 13 | Ancestors can affect my fortunes during bon | お盆の時期には、先祖は私の運命に影響を与えることができる。 |
| 14 | My ancestors would be bothered if I didn’t pray to them during bon | お盆の時期に私がお祈りをしないと、先祖が気にするだろう。 |
| 15 | Ancestors do not return during bon (R) | お盆の時期に、先祖の魂が家に帰ってくるわけではない。 |
| 16 | I don't feel ancestors are around me during bon (R) | お盆の時期だからといって、先祖の魂が自分の周りにいる気はしない。 |
| 17 | When I pray during bon, my ancestors aren’t listening (R) | お盆の時期に私が祈ったとしても、先祖がそれを聞くわけではない。 |

The instructions and response scale were also developed during this focus group session. Instructions to participants were “以下のそれぞれの文は、あなた自身の考えにどのくらい当てはまりますか。賛成または反対の度合いでお答えください。（注）文中に登場する「先祖」とは、あなたの両親や祖父・祖母、兄弟姉妹、子ども、あるいはそれ以前の人々を含む、すでに亡くなった親族を指します。” (How well does each of the following statements reflect your own thoughts? Please answer in favour or disagreement. Note: In the following questions “ancestors” refers to your parents, grandparents, siblings, children, and other relatives who have passed away, possibly including even those before these [as in older ancestors]). Participants in the focus group thought it was important that ‘ancestors’ be defined in our scale instructions so that respondents knew exactly what we were asking about. The response scale was a 4-point Likert scale from 1 – “全くそう思わない” (strongly disagree), to 4 – “強くそう思う” (strongly agree).

# Validation of the Bon Belief Scale

## Participants

To validate our scale, we surveyed 151 Japanese users of the online freelance and crowdsourcing site Lancers. Sample demographics were 43% female and 57% male, with ages ranging from 18 to 73 with a mean age of 42.

Missing data from 19 participants was imputed using MICE [7]

## Materials

Alongside the Bon scale that we developed in this study, we also measured responses to the Supernatural Belief Scale (SBS) [8], Nishiwaki’s belief scale [9], the Big Five Inventory-2 [10], the Portrait Values Questionnaire-RR (PVQ-RR) [11], and two behavioural measures (frequency of grave cleaning, and returning to the family home for Bon), which we used to assess convergent validity for our belief scale.

The Supernatural Belief Scale developed by Jong, and Halberstadt [8], has an existing official Japanese version, as mentioned earlier in the text, that was slightly modified to suit a Japanese context. It was measured on a 5-point Likert scale, from -2 to 2 where -2 means “非常に反対” (Very much the opposite) and 2 means “非常に賛成” (Very much agree). Items included “霊的で全知全能の存在を神と呼ぶことがある。” (There exist all-powerful, all-knowing beings whom we might call *kami*) and “守護神や悪魔のような善悪のある霊的な存在がいる。” (There exist spiritual beings, who might be good or evil, such as *shugoshin* or *akuma*).

Nishiwaki’s belief scale [9] is a scale developed in Japan by Japanese religion and psychology scholar, Ryo Nishiwaki. It aims to measure belief in the supernatural in three facets, beliefs in the divine, beliefs in religion in general, and beliefs in mystic forces in life and natural phenomenon. The first of these, beliefs in the divine, includes items such as “私は、神や仏の助けを受けていると感じることがある。” (I feel God or Buddha helps me), and “神や仏は、いつも私を見ていると思う。” (I believe or God or Buddha is always watching me). Beliefs in religion in general is made up of items such as “宗教は、人に助け合いの心をもたせ、良い人間関係をむすぶことができるようにしてくれると思う。” (I think religion gives a spirit of mutual aid and helps develop good relationships), and “宗教は苦しみをやわらげ、心をいやしてくれると思う。” (I think religion relieves my mental distress and heals my heart). The final facet, beliefs in mystic forces, includes the items “この宇宙のあらゆるの（星、人、動物、山々、森など）には何か霊的なものが宿っていると思う。” (I believe spiritual things dwell everywhere in the universe—such as the stars, humans, animals, mountains, and forests and so on), and “人間の考えをこえた力をもつような、何か神秘的な存在があると思う。” (I believe there is some mysterious entity which has power beyond our thoughts). These items are measured on a 5-point Likert scale from 1 “全く当てはまらない” (Completely disagree), to 5 “とても当てはまる” (Completely agree).

The Big Five Inventory-2-S [10] is a scale developed to measure the big five personality traits, Extraversion, Agreeableness, Conscientiousness, Negative emotionality/Neuroticism, and Open-mindedness. It was translated into Japanese by the International Situations Project (n.d.). Items in the negative emotionality subscale include “気難しく、簡単に感情的になる” (Is temperamental, gets emotional easily). The conscientiousness subscale includes items such as “粘り強く、作業が終わるまで働く” (Is persistent, works until the task is finished). Items in the open-mindedness subscale include “独自の、新しいアイディアを出せる” (Is original, comes up with new ideas). The agreeableness subscale includes “人々に対して最善をつくす” (Assumes the best about people). Finally. The extraversion subscale includes items such as “活力にあふれている” (Is full of energy). The items in this scale are scored on a 5-point Likert scale from 1 “全く同意できない” (Disagree strongly), to 5 “とても同意する” (Agree strongly).

The Portrait Values Questionnaire-RR [11] assesses a person’s values, and three of these have been found to correlate consistently with religious belief, namely security, tradition, and conformity [12-14]. As the full scale is 54 items, we chose to only include the relevant subscales, bringing it down to 15. Items in these subscales included, “この人にとっては、病気を予防し、健康を守ることが非常に重要である” (It is very important to him to avoid disease and protect his health.) (Security), “この人にとっては、ルールや規則には、決して違反しないことが重要である” (It is important to him never to violate rules or regulations) (Conformity), and “この人にとっては、伝統的な価値観や考え方を持ち続けることが重要である” (It is important to him to maintain traditional values and ways of thinking) (Tradition). These items were measured on a 6-point Likert scale from 1 “まったく似ていない” (Not at all similar to me) to 6 “とても似ている” (Very similar to me). It was translated into Japanese by Manabe [15].

## Results

An exploratory factor analysis on our scale was run to assess the possible dimensionality. A scree test showed one factor explaining a large amount of variance, eigenvalue = 10.22. The single factor explained 60% of the variance and adding a second factor (eigenvalue = 0.74) only added a further 5% of the variance. Given that there was a strong first factor and the second factor had an EV below 1, we continued with a single factor solution. As can be seen in Table 2, all items loaded strongly onto a single factor. Items all had means hovering around or just above the mid-way point (Min = 1.748, Max = 3.040), and standard deviations were all between 0.76 and 0.94.

| **Supplemental Table 1** | | | |  |
| --- | --- | --- | --- | --- |
|  | **Item Statistics** | | | **Single Factor Analysis** |
|  |  | **Mean** | **SD** | **Factor 1** |
| 1 | During bon, my ancestors travel home | 2.556 | 0.861 | 0.86 |
| 2 | I can talk to ancestors during bon | 2.106 | 0.865 | 0.79 |
| 3 | I feel like ancestors are watching me during bon | 2.305 | 0.856 | 0.87 |
| 4 | I feel ancestors are around me during bon | 2.331 | 0.892 | 0.88 |
| 5 | Ancestors leave again at the end of bon | 2.457 | 0.929 | 0.81 |
| 6 | I feel like ancestors are physically close to me during bon | 2.113 | 0.853 | 0.85 |
| 7 | I feel like ancestors are psychologically close during bon | 2.311 | 0.939 | 0.86 |
| 8 | It is important to pray to ancestors during bon. | 3.040 | 0.848 | 0.67 |
| 9 | Cleaning the family grave shows returning ancestors they are cared for | 2.781 | 0.886 | 0.71 |
| 10 | Ancestors reside in the butsudan during bon | 2.179 | 0.841 | 0.76 |
| 11 | My ancestors listen when I pray during bon | 2.351 | 0.850 | 0.77 |
| 12 | It is important to provide offerings for ancestors during bon | 2.695 | 0.841 | 0.68 |
| 13 | Ancestors can affect my fortunes during bon | 1.748 | 0.768 | 0.58 |
| 14 | My ancestors would be bothered if I didn’t pray to them during bon | 2.205 | 0.835 | 0.73 |
| 15 | Ancestors do not return during bon (R - recoded) | 2.497 | 0.916 | 0.83 |
| 16 | I don't feel ancestors are around me during bon (R - recoded) | 2.444 | 0.884 | 0.77 |
| 17 | When I pray during bon, my ancestors aren’t listening (R - recoded) | 2.444 | 0.877 | 0.67 |
|  |  |  | |  |

*Note: Omitted values had loadings under 0.30*

Internal reliability was good (α = .96), and, checking our new scale’s correlations with the other variables that we included in the study we found good convergent validity. The new scale had significant moderate positive correlations with both Jong and Halberstadt’s [8] SBS (r = .556, *p* < .001), and Nishiwaki’s [9] belief scale (r = .66, *p* < .001) which suggests that our scale is measuring a similar concept to both of these other belief scales. These scales both measure more general belief in concepts such as the existence of gods, spirits, and supernatural forces so this result was in line with our expectations.

We expected our scale to have a positive relationship with the Big Five factors agreeableness and conscientiousness (Saroglou, 2010; Gebauer et al., 2014; Robbins, Francis, McIlroy, Clarke, and Pritchard, 2010). Our scale did have a significant weak positive correlation with agreeableness (r = .19, *p* = .020) but no significant relationship with conscientiousness (for comparisons with previous studies, see [16-18]. We also found a weak negative relationship with negative emotionality (r = -.17, *p* = .04) which, while not particularly common, has been found in previous literature [19, 20].

We also checked our measure against three of the Schwartz values as measured by the Portrait Values Questionnaire [11], security, conformity, and tradition, which have found to be related to religiosity in multiple cultural contexts [12, 13] and in a 15 country meta-analysis by Saraoglou, Delpierre, and Dernelle [14]. Our measure had a significant weak positive correlation with conformity (r = .29, *p* < .001), and significant moderate positive correlation with tradition (r = .41, *p* < .001).

Additionally, our measure had significant weak positive correlations with the behavioural measure of grave cleaning (r = .17, *p* = .03), and returning to the family home during Bon (r = .30, *p* < .001). We suspect that the weakness of these correlations is due to the normative aspects of these activities, where even those who do not believe are expected to perform them, along with the access factor, i.e., that some who would like to perform these activities because of their beliefs are not able to because of where they live or work.

In order to make the scale appropriate for use in our field study, we shortened the scale to the five highest loading items from the exploratory factor analysis, items 1, 3, 4, 6 and 7. After assessing the items selected, we decided to switch items 6 and 7 for items 2 and 5 (the next highest loading, bar item 15), as items 6 and 7 were too similar to item 4. Though item 15 was loading higher than item 2, we decided not to include it, as it was only a negative coded version of item 1 and recent research has suggested that negative coding is not helpful and may confuse participants [21]. After finalising this shortened scale, we repeated the test of internal reliability (α = .94), and convergent validity. The only changes in correlations between our scale and the other measures when using the shortened scale were that the weak correlations between belief and negative emotionality, and belief and grave cleaning became non-significant. As these had been weak correlations even with the original 17 item scale, we considered this change insignificant and decided the shortened scale would be sufficient for use in study 3.

| **Supplemental Table 2** | | |
| --- | --- | --- |
| **Bon Belief Scale – Shortened** | | |
|  | **English Items** | **Japanese Items** |
| 1 | During bon, my ancestors travel home | お盆の時期には、先祖の魂が家に帰る。 |
| 2 | I can talk to ancestors during bon | お盆の時期には、先祖の魂に話しかけることができる。 |
| 3 | I feel like ancestors are watching me during bon | お盆の時期には、先祖が自分を見ているような気がする。 |
| 4 | I feel ancestors are around me during bon | お盆の時期には、先祖の魂が自分の近くにいるような気がする。 |
| 5 | Ancestors leave again at the end of bon | お盆が終わると、先祖の魂はまた帰って行く。 |
|  |  | |

**References**

1. Hori, I. (1959). Japanese folk-beliefs. American Anthropologist, 61, 405-424.
2. Yanagita, K. (1970). About our ancestors: The Japanese family system (F. H. Mayer, Trans.). Tokyo: Japan Society for the Promotion of Science.
3. Berentsen, J. M (1985). Grave and Gospel. (Beihefte Der Zeitschrift Für Religions- und Geistesgeschichte; 30). Leiden, Netherlands: Brill.
4. Hendry, J., & Webber, J. (Eds.). (1998). Interpreting Japanese society: anthropological approaches. Retrieved from https://ebookcentral.proquest.com
5. Mayer, F. H. (1989). The Calendar of Village Festivals: Japan. Asian Folklore Studies, 48(1), 141-47.
6. Ooms, H. (1967). The Religion of the Household: A Case Study of Ancestor Worship in Japan. Contemporary Religions in Japan, 8(3/4), 201–333.
7. Wulff, J. & Ejlskov, L. (2017) Multiple Imputation by Chained Equations in Praxis: Guidelines and Review. The Electronic Journal of Business Research Methods 15(1), 41-56.
8. Jong, J., & Halberstadt, J. (2016). Death anxiety and religious belief: an existential psychology of religion. London, UK: Bloomsbury Academic.
9. Nishiwaki, R (2004). Nihonjin no shukyoteki shizenkan: Ishiki chosa niyoru jisshouteki kentou [A religious view of nature in Japan]. Kyoto, Japan: Minerva.
10. Soto, C.J., & John, O.P. (2017). The next Big Five Inventory (BFI-2): Developing and assessing a hierarchical model with 15 facets to enhance bandwidth, fidelity, and predictive power. Journal of Personality and Social Psychology, 113, 117-143.
11. Schwartz, Shalom H. et al.(2012). Refining the Theory of Basic Individual Values. Journal of Personality and Social Psychology, 103(4). 663-688
12. Cukur, C. S., de Guzman, M. R. T., & Carlo, G. (2004). Religiosity, values, and horizontal and vertical individualism-collectivism: A study of turkey, the united states, and the philippines. The Journal of Social Psychology, 144(6), 613-34. doi:10.3200/SOCP.144.6.613-634
13. Aarnio, K., & Lindeman, M. (2007). Religious people and paranormal believers: Alike or different? Journal of Individual Differences, 28(1), 1-9. doi:10.1027/1614-0001.28.1.1
14. Saroglou, V., Delpierre, V., & Dernelle, R. (2003). Values and Religiosity: A Meta-Analysis of Studies Using Schwartz's Model. Personality and Individual Differences, 37(4), 721-734. doi:10.1016/j.paid.2003.10.005.
15. Manabe, K. (2018). <Research Note> Methodological examination of the Schwartz Values Survey. Kwansei Gakuin University Sociology Department Bulletin (129), 75-94. https://kwansei.repo.nii.ac.jp/?action=pages_view_main&active_action=repository_view_main_item_detail&item_id=27443&item_no=1&page_id=30&block_id=85
16. Saroglou, V. (2010). Religiousness as a Cultural Adaptation of Basic Traits: A Five-Factor Model Perspective. Personality and Social Psychology Review, 14(1), 108–z125. doi:10.1177/1088868309352322
17. Gebauer, J. E., Bleidorn, W., Gosling, S. D., Rentfrow, P. J., Lamb, M. E., & Potter, J. (2014). Cross-cultural variations in big five relationships with religiosity: A sociocultural motives perspective. Journal of Personality and Social Psychology, 107(6), 1064-1091. doi:10.1037/a0037683
18. Robbins, M., Francis, L., McIlroy, D., Clarke, R., & Pritchard, L. (2010). Three religious orientations and five personality factors: An exploratory study among adults in england. Mental Health, Religion & Culture, 13(7-8), 771-775. doi:10.1080/13674676.2010.519468
19. Saroglou, V. (2002). Religion and the five factors of personality: A meta-analytic review. Personality and Individual Differences, 32(1), 15–25. <https://doi.org/10.1016/S0191-8869(00)00233-6>
20. Khoynezhad, G., Rajaei, A. R., & Sarvarazemy, A. (2012). Basic religious beliefs and personality traits. Iranian journal of psychiatry, 7(2), 82–86.
21. van Sonderen, E., Sanderman, R., & Coyne, J. C. (2013). Ineffectiveness of reverse wording of questionnaire items: let's learn from cows in the rain. PloS one, 8(7), e68967. doi:10.1371/journal.pone.0068967
